# The COVID-19 pandemic: the effect on airway Management in non-COVID emergency patients

**DOI:** 10.1186/s12873-021-00491-7

**Published:** 2021-08-28

**Authors:** Onlak Ruangsomboon, Phetsinee Boonmee, Akarin Nimmannit

**Affiliations:** 1 Department of Emergency Medicine, Faculty of Medicine, Siriraj Hospital, Mahidol University, 2 Wanglang Road, Bangkoknoi, Bangkok, 10700 Thailand; 2 Department of Research, Faculty of Medicine, Siriraj Hospital, Mahidol University, 2 Wanglang Road, Bangkoknoi, Bangkok, 10700 Thailand

**Keywords:** COVID-19, Airway management, Non-invasive ventilation, High-flow nasal cannula

## Abstract

**Background:**

During the COVID-19 outbreak, healthcare providers might have avoided droplet/aerosol-generating procedures, such as non-invasive ventilation (NIV) and high-flow nasal cannula (HFNC) due to the concern of themselves being infected. We hypothesized that this change of practice could have also occurred to other non-COVID-infected patients in the Emergency Department (ED).

**Methods:**

A retrospective analytic study was conducted in the ED of Siriraj Hospital, Bangkok, Thailand, including adult patients presenting with signs and symptoms of respiratory distress between 1 March and 30 April 2020 (the COVID period). A comparison group using the same inclusion criteria was retrieved from 1 March to 30 April 2019 (the pre-COVID period). The primary outcome was rate of NIV and HFNC use. The secondary outcomes were rate of intubation, failure of NIV and HFNC, complications, and mortality.

**Results:**

A total of 360 and 333 patients were included during the pre-COVID and COVID periods, respectively. After adjusting for baseline differences, patients in the COVID period were less likely to receive either NIV or HFNC than the pre-COVID period (adjusted OR 0.52 [95%CI 0.29–0.92]). Overall, intubation rate was similar between the two study periods. However, patients in respiratory distress with pulmonary edema had a relatively higher intubation rate in the COVID period. There were higher failure rates of NIV and HFNC, more infectious complications, and a higher rate of mortality in the pre-COVID period.

**Conclusion:**

During the COVID-19 pandemic, the overall usage of NIV and HFNC in emergency non-COVID patients decreased. Although not affecting the overall intubation rate, this change of practice could have affected some groups of patients. Therefore, treatment decisions based on a balance between the benefits to the patients and the safety of healthcare providers should be made.

**Supplementary Information:**

The online version contains supplementary material available at 10.1186/s12873-021-00491-7.

## Background

Since late December 2019, a number of patients in Wuhan, China suffered from a novel coronavirus [1]. To date, this coronavirus disease 2019 (COVID-19) has spread globally and has infected millions of people worldwide. Consequently, the World Health Organization (WHO) declared COVID-19 as a pandemic disease and a public health emergency of international concern in early March 2020 [2]. In Thailand, a southeast Asian country with 69.8 million inhabitants, confirmed COVID-19 cases emerged in mid-January and the epidemic spread rapidly during March and April 2020 with around 3000 confirmed cases and a 1.8% mortality rate by early May 2020 [3].

COVID-19 is a respiratory tract infection, causing a wide range of disease severity. Although most of the infected patients present with mild or uncomplicated viral upper respiratory tract complaints, some patients who develop the severe form may present with respiratory distress and hypoxemia needing oxygen supplement and respiratory support [2, 4]. The use of noninvasive ventilation (NIV) and high-flow oxygen via nasal cannula (HFNC) for this condition has been controversial. Both NIV and HFNC have shown beneficial effects for hypoxemic respiratory failure [5–7], and recent reports in COVID-19 patients have confirmed these benefits [4, 8, 9]. However, the possibility of both respiratory interventions spreading droplets or creating aerosols transmitted to healthcare providers has caused much debate and concern. A study by Hui et al. proved that dispersion is minimal if the mask interface is tightly sealed [10]. Moreover, no guidelines are against their use if healthcare providers wear appropriate personal protective equipment (PPE), preferably in a negative pressure area [2, 11]. However, many physicians are still reluctant to use noninvasive airway procedures. With large numbers of healthcare providers infected during the COVID-19 outbreak [12], the issue of potential aerosol-generating procedures was raised, consequently causing a trend to avoid NIV, HFNC, and nebulization. Interestingly, this is not only the case with COVID-19 patients, but also with non-COVID patients, especially those presenting with respiratory distress in the emergency setting, in whom COVID-19 status has not yet been confirmed.

There have been many reports elaborating on the impact of COVID-19 on other non-COVID patients [13–16]. Multiple centers have observed that the rate of admission due to other common emergency conditions, such as acute coronary syndrome and stroke, has dropped dramatically [14, 15]. Patients eventually visit the hospital when their conditions have progressively worsened. Some treatment options are not available or have been delayed until after a negative COVID-19 status confirmation [13]. Patients presenting to the emergency department (ED) with respiratory distress may have different characteristics and severity of dyspnea at presentation in the COVID-19 pandemic period. In the ED with limited PPE resources and negative pressure rooms, decisions on respiratory support options may have also been altered because of healthcare providers’ concerns over potential aerosolization of virus particles and the risk of themselves becoming infected, even from patients with low clinical suspicion of COVID-19.

We conducted this retrospective analytical study to examine the characteristics, the respiratory interventions given, and outcomes of patients presenting to the ED with respiratory distress in the COVID-19 pandemic period compared with those in a pre-COVID period.

## Methods

### Study design and settings

This study was a retrospective cohort study including two groups from two different time periods; one from 1 March to 30 April 2019 (the pre-COVID period) and the other from 1 March to 30 April 2020 (the COVID period). The study was conducted at the ED of Siriraj Hospital, Mahidol University, which is the largest tertiary university hospital in Bangkok, Thailand. The ED has an annual visit rate of over 20,000 Emergency Severity Index triage level 1–2 patients alone. The Siriraj Institutional Review Board approved the study protocol and waived the need for informed consent. The study protocol was performed in accordance with the Declaration of Helsinki and is reported according to the STROBE guideline [17]. This study was supported by Siriraj Research Development Fund (Managed by Routine to Research: R2R).

Due to the COVID-19 outbreak, the Thai government imposed a night curfew starting on 3 March 2020 until at least 31 May 2020. Siriraj Hospital had postponed all possible outpatient appointments and elective procedures and surgery. All patients had to pass the COVID-19 triage area staffed by triage nurses before entering the hospital. Patients suspected of having COVID-19 from this primary triage were transferred to be evaluated by infectious disease (ID) specialists. The ID specialists determined which patients were classified as Patient Under Investigation (PUI) for COVID-19, and the PUIs were transferred to a designated COVID-19 ward for specimen collection. Patients who passed the initial screening were sent to the outpatient clinic or the ED according to their triage level. If an ED physician suspected a patient of having a COVID-19 infection during secondary triage in the ED, an ID specialist would be consulted to determine the patient’s PUI status.

### Selection of participants

We included adult patients aged at least 18 years old who presented to the ED with signs and symptoms respiratory distress. This was defined as any complaints of respiratory or chest discomfort, and at least one of the following signs of respiratory distress; a respiratory rate > 24 breaths/min, a pulse oximetry reading (SpO_2_) at room air of less than 95%, and signs of increased work of breathing, such as accessory muscle use, as recorded by the ED physicians. Pregnant patients were excluded because they differ physiologically from non-pregnant patients and are usually considered contraindicated for NIV.

### Study protocol

We retrospectively assessed medical records of all ED patients for eligibility between 1 March and 30 April 2020. This was the period during which the COVID-19 outbreak was the most severe in Thailand, and we referred to this as the COVID period. We also assessed all ED patients during 1 March and 30 April 2019 (the pre-COVID period) to acquire a historic control group with the least different characteristics to the patients in the COVID period, regarding seasonal and epidemiological factors.

We extracted the following data from their medical records: age, sex, initial vital signs (temperature, heart rate, respiratory rate, blood pressure and pulse oximetry), baseline status and underlying diseases, diagnosis, details of oxygen supplementation and respiratory interventions in the ED and during the hospital stay, complications, and outcomes. Primary diagnoses were pneumonia defined as community-, hospital-acquired or healthcare-associated pneumonia, obstructive lung conditions defined as chronic obstructive pulmonary disease (COPD) or asthma, and conditions with pulmonary edema defined as congestive heart failure and volume overload.

### Outcome measures

The primary outcome was the rate of non-invasive airway equipment (NIV and HFNC) use. The secondary outcomes were intubation rate, complications such as failure of non-invasive oxygen or ventilatory support, hospital-associated pneumonia (HAP), ventilator-associated pneumonia (VAP), and septic shock that occurred after 48 h of ED admission, ED and hospital length of stay, and 30-day and in-hospital mortality. Respiratory interventions initiated within 30 min of ED arrival were considered initial intervention while those initiated after 30 min were deemed rescue interventions. Failure of NIV and HFNC was defined by any escalation to a more invasive airway procedure.

### Statistical analyses

Descriptive statistics are presented as frequency (%) for categorial variables, mean (SD) for continuous variables with parametric distribution, and median (Q1, Q3) for continuous variables with nonparametric distribution. Continuous variables were assessed for parametric distribution by histograms, Q-Q plots, and the Shapiro Wilk test. Risk ratios of respiratory intervention use during the ED visit between the pre-COVID and COVID periods were estimated by inverse probability of treatment (defined as exposure of COVID period) weighted (IPTW) analysis using a propensity score. This propensity score was calculated using a multivariable logistic regression with time period as the dependent variable with age, gender, Charlson comorbidity index (CCI), initial SpO_2_/FiO_2_ ratio, respiratory rate, and mean arterial pressure as the independent variables. These variables were chosen as prognostic factors of NIV and HFNC. IPTW weights were calculated to assess the average treatment effect. Balance statistics for the prognostic factors were calculated as standardized mean differences before and after IPTW weighting. Balance was considered acceptable if the standardized mean difference was > +/− 0.1. If extreme weights were detected, 99th percentile trimmed analysis would also be performed. The outcome was calculated as an adjusted odds ratio using a generalized estimating equation to account for correlated data. Risk ratios of mortality outcomes were estimated by multivariable logistic regression adjusting for age, gender, and CCI. A priori subgroup analyses of patients without do-not-intubate (DNI) status were also performed.

We also performed secondary analyses. Initial severity based on respiratory rate and SpO_2_/FiO_2_ ratio was stratified by age group and diagnosis as well as by age and respiratory intervention to compare initial severity between patients in the pre-COVID and COVID periods. Endotracheal intubation and mortality rates in the subgroup of patients stratified by diagnosis were also compared between the pre-COVID and COVID periods. We performed analyses using SPSS 18.0 (IBM Corp., Chicago, IL) and R (version 3.2.1; R Foundation for Statistical Computing, Vienna, Austria).

## Results

### Characteristics of study subjects

**Fig. 1:**
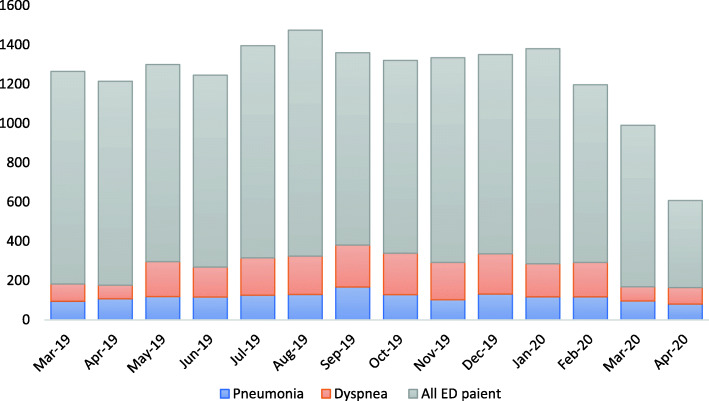
Number of patients visiting the ED during March 2019 and April 2020. Note: Pneumonia = community-, hospital-acquired or healthcare associated pneumonia. Dyspnea = patients visited the ED with acute respiratory distress. Abbreviation: ED, emergency department

During the COVID period, the overall ED visit rate was lower compared to the pre-COVID period, as well as the previous 10 months (Fig. 1). However, the proportion of patients presenting with acute respiratory distress during the COVID period was higher than that of the pre-COVID period (20.7 and 14.5%). Of 333 patients in the COVID period, 19 (5.7%) were considered PUIs during secondary triage in the ED, but no patients tested positive for COVID-19. The characteristics of participants of the two study groups are presented in Table 1. Patients who visited the ED in the pre-COVID period were slightly older with higher mean CCI compared to patients in the COVID period. Initial vital signs were similar despite a higher mean mean arterial pressure and lower mean oxygen saturation and SpO_2_/FiO_2_ ratio in the COVID period. A similar proportion of patients in both periods had primary diagnoses of pneumonia, obstructive lung conditions, and conditions with pulmonary edema.

**Table 1:** Comparison of characteristics of patients with acute respiratory distress

Characteristics	pre-COVID (***n*** = 360)	COVID (***n*** = 333)	***P***-value
Age, year	71.3 + 14.8	68.7 + 16.8	**0.03**
Sex (male)	191 (53.1)	168 (50.5)	0.49
Underlying disease			
Diabetes mellitus	116 (32.2)	105 (31.5)	0.85
Hypertension	238 (66.1)	166 (49.8)	**< 0.0001**
Old cerebrovascular disease	85 (23.6)	46 (13.8)	**0.001**
Chronic kidney disease or End stage kidney disease	85 (23.6)	69 (20.7)	0.36
Chronic obstructive pulmonary disease	76 (21.6)	50 (15.0)	**0.04**
Asthma	26 (7.2)	39 (11.7)	**0.04**
Coronary artery disease	75(20.8)	46(13.8)	**0.02**
Cancer	63 (17.5)	69 (20.7)	0.28
Do-not-intubate status	107 (29.7)	82 (24.6)	0.13
Charlson comorbidity index	5.5 + 2.8	5.0 + 2.6	**0.01**
Initial vital signs			
Body temperature, ^o^C	37.3 + 0.8	37.2 + 0.9	0.14
Pulse rate, beats/min	102.8 + 23.3	104.7 + 25.9	0.32
Mean arterial pressure, mmHg	97.9 + 22.1	104.1 + 23.5	**< 0.0001**
Respiratory rate, breaths/min	32.1 + 66	32.4 + 6.0	0.49
Oxygen saturation, %	92.2 + 6.7	90.4 + 9.7	**0.003**
SpO_2_/FiO_2_ ratio	438 (395, 457)	428 (383, 452)	**0.09**
Diagnosis			
Community acquired pneumonia	152 (42.2)	139 (41.7)	0.90
Hospital acquired pneumonia/ health-care associated pneumonia	51 (14.2)	39 (11.7)	0.34
Chronic obstructive pulmonary disease	67 (18.6)	42 (12.6)	**0.03**
Asthma	20 (5.6)	34 (10.2)	**0.02**
Congestive heart failure	66 (18.3)	67 (20.1)	0.55
Volume overload	9 (2.5)	20 (6)	**0.02**
Pleural effusion	0 (0)	11 (3.3)	**0.001**
Pulmonary embolism	0 (0)	4 (1.2)	**0.04**
Chronic lung disease	11 (3.0)	9 (2.7)	0.23
Management			
Bronchodilator			
Nebulizer	200 (55.6)	57 (17.1)	**< 0.0001**
Metered-dose inhaler	–	48 (14.4)	
Initial respiratory intervention			
Oxygen cannula	140 (38.9)	126 (37.8)	0.112
Oxygen mask with reservoir bag or collar	99 (27.5)	84 (25.2)	
High-flow nasal cannula	5 (1.4)	2 (0.6)	
Non-invasive ventilator	10 (2.8)	5 (1.5)	
Endotracheal tube	27 (7.5)	23 (6.9)	
Rescue intervention			
High-flow nasal cannula	10 (2.8)	4 (1.2)	0.33
Non-invasive ventilator	12 (3.3)	10 (3.0)	
Endotracheal tube	28 (7.8)	26 (7.8)	
Complications			
Hospital acquired pneumonia (HAP)	16 (4.4)	10 (3.0)	**0.002**
Ventilator associated pneumonia (VAP)	19 (5.3)	9 (2.7)	
Septic shock	17 (4.7)	12 (3.6)	
HAP, VAP and shock	17 (4.7)	5 (1.5)	
ED disposition			
Admit general ward/ICU	178 (49.4)	165 (49.5)	**0.001**
Admit isolation ward for COVID-19	–	18 (5.4)	
Observation room	66 (18.3)	61 (18.3)	
Discharge	63 (17.5)	51 (15.3)	
Refer	26 (7.2)	23 (6.9)	
Death	27 (7.5)	15 (4.5)	
Outcome			
ED length of stay, hour	6.5 (4, 14.8)	5 (3, 10)	**< 0.0001**
Hospital length of stay, day	4 (1, 9)	4 (1, 7)	0.13

Note: Data presented as n (%), mean + SD or median (Q1, Q3) and compared using the Chi-squared or Fisher’s Exact test for categorical data and independent t-test or Mann-Whitney U test for continuous data. A p-value < 0.05 was considered significant. Pre-COVID = patients included during March–April 2019, COVID = patients included during March–April 2020

Abbreviations: ED, emergency department; ICU, intensive care unit; COVID, coronavirus disease

### Respiratory interventions, complications, and outcomes

Standardized mean differences of propensity score covariates were acceptably balanced at values of less than +/− 0.1 after IPTW weighting (Table S1). Rate of overall use of NIV or HFNC was 21 (6.3%) and 36 (10%) in the COVID and pre-COVID periods, respectively. After adjusting for baseline differences, patients in the COVID period had significantly lower odds of receiving either NIV or HFNC (adjusted OR 0.52 [0.29, 0.92]) (Table 2). They also had a trend towards lower odds of receiving initial treatment with NIV or HFNC (adjusted OR 0.40 [95%CI 0.16, 1.00]). However, this result is compatible with no difference between the pre-COVID and COVID period due to the 95%CI. The overall intubation rate was also similar between the two periods (Table 2).

**Table 2:** Estimated effects of COVID period on the use of respiratory interventions and mortality in patients with acute respiratory distress comparing the COVID with the pre-COVID period

Outcomes	Pre-COVID (*n* = 360)		COVID (*n* = 333)		Adjusted OR (95%CI)
	n	% (95%CI)	n	% (95%CI)	
**Respiratory interventions**					
					Inverse PS score weighted
Initial NIVHFNC use	15	4.2 (2.3, 6.8)	7	2.1 (0.8, 4.3)	0.40 (0.16, 1.00)
NIVHFNC use	36	10.0 (7.1, 13.6)	21	6.3 (3.9, 9.5)	**0.52 (0.29, 0.92)**
^a^ Intubation	55	21.7 (16.8, 27.3)	49	19.5 (14.8, 24.9)	0.94 (0.46, 1.32)
Pneumonia	34	32.1 (23.3, 41.8)	29	26.4 (18.4, 35.6)	0.76 (0.42, 1.37)
COPD/Asthma	8	8.6 (3.8, 16.2)	7	10.0 (4.1, 19.5)	1.18 (0.41, 3.42)
Pulmonary edema	10	14.1 (7.0, 24.4)	18	23.4 (14.5, 34.4)	1.86 (0.79, 4.36)
**Mortality**					
					Multivariable logistic regression
Non-survivor within 30 days	89	24.7 (20.4, 29.5)	43	12.9 (9.5, 17.0)	**0.50 (0.33, 0.76)**
Pneumonia	75	37.4 (30.8, 44.4)	36	19.7 (14.1, 26.3)	**0.48 (0.29, 0.77)**
COPD/Asthma	11	11.8 (6.2, 19.6)	2	2.6 (0.3, 9.2)	-^b^
Pulmonary edema	3	4.0 (0.8, 11.2)	5	5.8 (1.9, 13.0)	-^b^
In-hospital mortality	72	20.0 (16.0, 24.5)	40	12.0 (8.7, 16.0)	**0.62 (0.40, 0.95)**

Note:- 95%CI for proportions were calculated by the Clopper Pearson Exact method. Pre-COVID = patients included during March–April 2019, COVID = patients included during March–April 2020. For respiratory interventions, inverse propensity score (PS) weighted analyses adjusted for age, gender, Charlson comorbidity index, respiratory rate, mean arterial pressure, and SpO_2_/FiO_2_ ratio. For mortality outcomes, multivariable logistic regression adjusted for age, gender, and Charlson comorbidity index. Pneumonia = community-, hospital-acquired or healthcare associated pneumonia. Pulmonary edema = congestive heart failure or volume overload. Bold fonts are statistically significant results. A non-null 95% confidence interval was considered significant

Abbreviation: COPD, chronic obstructive pulmonary disease; OR, odds ratio; CI, confidence interval; COVID, coronavirus disease

^a^ Excludes patients with do-not-intubate orders: pre-COVID period *n* = 253, COVID period *n* = 251. Odds ratios of subgroups are crude ORs

^b^ Regression not performed due to insufficient events per variable

Failure rates of NIV and HFNC were 27.3 and 13.3%, respectively, in the pre-COVID period, and 6.7 and 0%, respectively, in the COVID period. The rates of HAP, VAP, and septic shock were significantly higher in the pre-COVID period (Table 1). Patients in the pre-COVID period stayed in the ED longer than the COVID period (difference 1.5 h [95%CI 0.1–2.9]). They also had significantly higher 30-day and hospital mortality rates, especially those with pneumonia (Table 2). A subgroup of patients without DNI status in the COVID period had lower odds of receiving NIV or HFNC (adjusted OR 0.14 [0.10, 0.20]) and lower risk of developing 30-day mortality than those in the pre-COVID period (adjusted OR 0.30 [0.14, 0.64]).

**Fig. 2:**
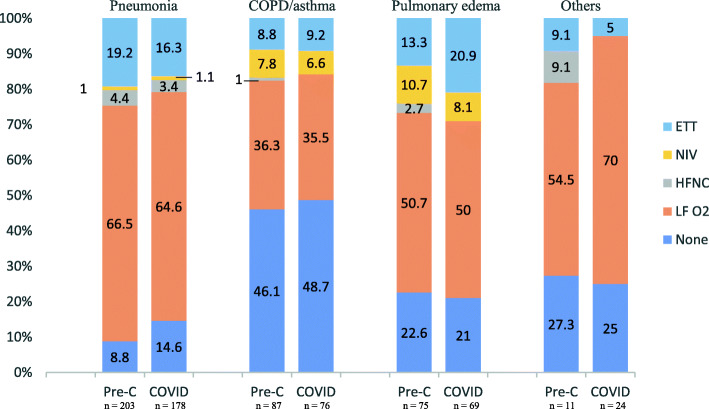
Respiratory interventions by diagnosis. Note: Pre-C group = patients included during March–April 2019, COVID group = patients included during March–April 2020. Pneumonia = community-, hospital-acquired or healthcare associated pneumonia, Pulmonary edema = congestive heart failure or volume overload. Abbreviations: COPD, chronic obstructive pulmonary disease; ETT, endotracheal intubation; NIV, non-invasive ventilation; HFNC, high-flow nasal cannula; LF O2, low flow oxygen consisting of standard nasal cannula, non-rebreather mask and collar mask

Also, a significantly lower number of patients had bronchodilator prescribed in the ED during the COVID period (31.5%) compared to the pre-COVID period (55.6%), and almost half of the prescribed bronchodilators in the COVID period were delivered by metered-dose inhaler (MDI) rather than standard nebulizer (Table 1). The comparison of respiratory interventions by each primary diagnosis is presented in Fig. 2. Patients with pneumonia in both periods had a similar rate of intubation and NIV. For patients with obstructive lung conditions and conditions with pulmonary edema, the use of NIV and HFNC was relatively lower in the COVID period. Additionally, more patients with pulmonary edema in the COVID period were intubated, however, it did not reach statistical significance (OR 1.86 [0.79, 4.36]) (Table 2).

### Secondary analyses

We analyzed and compared the severity at ED presentation based on the patients’ primary group of diagnoses. In the COVID period, patients with obstructive lung conditions had an overall higher mean respiratory rate but a comparable mean SpO_2_/FiO_2_ ratio. Patients with pulmonary edema had an overall lower mean SpO_2_/FiO_2_ ratio, especially in those aged less than 70 years, despite a similar mean respiratory rate to the pre-COVID period (Table 3). We further examined the patients’ initial severity based on their highest respiratory intervention. We found that, despite comparable initial mean SpO_2_/FiO_2_ ratio and respiratory rate, there was a trend towards lower PaO_2_/FiO_2_ ratio after intubation in the COVID period (difference −  55 [95%CI -115, 5.5]). They also had a longer median duration (hour) of intubation (difference 67 [95%CI 35, 99]). In addition, we found that elderly patients aged more than 70 years who were treated with HFNC in the COVID period were initially more severe, with a higher mean respiratory rate. NIV was also initiated at a lower mean SpO_2_/FiO_2_ ratio in this elderly subgroup (Table 4).

**Table 3:** Initial severity according to the primary diagnosis

Age group	Respiratory rate			SpO_**2**_/ FiO_**2**_ ratio		
	Pre-COVID	COVID	Difference (95%CI)	Pre-COVID	COVID	Difference (95%CI)
**Pneumonia**						
	*n* = 203	*n* = 178		*n* = 203	*n* = 178	
Overall	33.1 + 6.7	33.4 + 6.3	- 0.3 (−1.6, 1.0)	424 (381, 452)	424 (367, 452)	0.0 (−13.1, 13.1)
	*n* = 79	*n* = 81		*n* = 79	*n* = 81	
Age 18–69	32.3 + 7.1	33.9 + 6.2	- 1.6 (− 3.5, 0.6)	433 (390, 457)	419 (366, 452)	- 14.3 (− 35.4, 6.8)
	*n* = 47	*n* = 39		*n* = 47	*n* = 39	
Age 70–79	32.1 + 6.2	32.0 + 6.5	0.1 (− 2.6, 2.9)	424 (362, 457)	400 (294, 438)	- 23.8 (− 55.1, 7.5)
	*n* = 77	*n* = 58		*n* = 77	*n* = 58	
Age > 80	34.0 + 6.6	33.2 + 6.4	0.8 (− 1.4, 3.0)	419 (309, 443)	438 (398, 452)	19.0 (− 1.2, 39.3)
**Chronic obstructive pulmonary disease and asthma**						
	*n* = 102	*n* = 76		*n* = 102	*n* = 76	
Overall	29.8 + 5.2	31.4 + 5.3	**- 1.6 (− 3.2,-0.04)**	443 (424, 462)	448 (412, 457)	4.8 (− 5.4, 14.9)
	*n* = 42	*n* = 37		*n* = 42	*n* = 37	
Age 18–69	28.1 + 4.7	30.3 + 4.4	- 2.2 (− 4.6, 0.1)	452 (438, 457)	448 (414, 457)	- 4.8 (− 16.6, 7.1)
	*n* = 32	*n* = 22		*n* = 32	*n* = 22	
Age 70–79	31.5 + 5.3	32.1 + 6.7	- 0.6 (− 3.9, 2.7)	440 (421, 457)	442 (410, 457)	2.4 (− 19.5, 24.3)
	*n* = 28	*n* = 17		*n* = 28	*n* = 17	
Age > 80	29.4 + 5.2	32.2 + 5.2	- 2.8 (− 6.0, 0.2)	443 (298, 464)	452 (405, 462)	9.5 (− 47.7, 66.8)
**Pulmonary edema**						
	*n* = 75	*n* = 86		*n* = 75	*n* = 86	
Overall	32.4 + 6.9	31.5 + 5.2	0.9 (− 1.0, 2.8)	452 (419, 290)	426 (314, 457)	**- 26.2 (− 42.6, − 9.8)**
	*n* = 31	*n* = 40		*n* = 31	*n* = 40	
Age 18–69	33.1 + 7.5	32.0 + 5.7	1.1 (− 2.0, 4.2)	462 (438, 471)	426 (314, 457)	**- 35.7 (− 71.0, − 0.5)**
	*n* = 24	*n* = 21		*n* = 24	*n* = 21	
Age 70–79	31.6 + 7.4	30.4 + 4.5	1.2 (− 2.5, 4.9)	438 (390, 461)	428 (400, 452)	- 9.5 (− 43.2, 24.2)
	*n* = 20	*n* = 25		*n* = 20	*n* = 25	
Age > 80	32.4 + 5.2	31.7 + 4.9	0.7 (− 2.4, 3.7)	455 (424, 462)	419 (305, 452)	- 35.7 (− 84.1, 12.6)

Note: Data presented as mean + SD or median (Q1, Q3) and compared using mean or median differences, respectively. 95%CIs for nonparametric distributions estimated by × 1000 bootstrapping. A non-null 95% confidence interval was considered significant. Bold fonts are statistically significant results. Pre-COVID = patients included during March–April 2019, COVID = patients included during March–April 2020. Pneumonia = community-, hospital-acquired or healthcare associated pneumonia. Pulmonary edema = congestive heart failure or volume overload

Abbreviations: SpO_2_/FiO_2_, oxygen saturation/fraction of inspired oxygen; CI, confidence interval; COVID, coronavirus disease

**Table 4:** Initial severity according to type of respiratory intervention

Age group	Endotracheal tube			Non-invasive ventilation			High-flow nasal cannula		
	Pre-COVID	COVID	Difference (95%CI)	Pre-COVID	COVID	Difference (95%CI)	Pre-COVID	COVID	Difference (95%CI)
**Respiratory rate**									
	*n* = 55	*n* = 49		*n* = 22	*n* = 15		*n* = 15	*n* = 6	
Overall	34.8 + 7.3	33.8 + 6.5	1.0 (− 1.7, 3.7)	33.0 + 6.4	33.7 + 4.2	- 0.7 (− 4.5, 3.2)	32.8 + 8.8	40.3 + 6.0	- 7.5 (−16, 0.7)
	*n* = 23	*n* = 24		*n* = 7	*n* = 7		*n* = 9	*n* = 2	
Age < 70	34.1 + 7.2	33.7 + 6.0	0.4 (−3.5, 4.3)	32.3 + 8.5	32.6 + 3.0	- 0.3 (− 8.3, 7.7)	32 (24, 36)	^a^ 32, 40	–
	*n* = 32	*n* = 25		*n* = 15	*n* = 8		*n* = 6	*n* = 4	
Age > 70	35.4 + 7.5	34.0 + 7.1	1.4 (−2.5, 5.3)	33.3 + 5.5	34.5 + 5.1	- 1.2 (− 6.1, 3.6)	33.0 + 6.4	42.5 + 7.6	**- 9.5 (− 19, − 0.4)**
**SpO**_**2**_**/ FiO**_**2**_ **ratio**									
	*n* = 55	*n* = 49		*n* = 22	*n* = 15		*n* = 15	*n* = 6	
Overall	390 (306, 433)	390 (303, 438)	0 (− 40, 40)	424 (381, 448)	376 (294, 448)	- 48 (− 150, 55)	400 (352, 452)	398 (232, 419)	- 2 (− 170, 165)
	*n* = 23	*n* = 24		*n* = 7	*n* = 7		*n* = 9	*n* = 2	
Age < 70	419 (380, 457)	369 (287, 424)	- 50 (− 121, 21)	414 (165, 429)	429 (371, 457)	- 15 (− 171, 200)	405 (357, 424)	^a^ 87.8, 419	–
	*n* = 32	*n* = 25		*n* = 15	*n* = 8		*n* = 6	*n* = 4	
Age > 70	374 (166, 429)	410 (347, 448)	36 (− 43, 114)	438 (395, 448)	301 (264, 433)	**- 137 (− 248, − 26)**	400 (352, 452)	397 (313, 433)	- 3 (− 138, 133)

Note: Data presented as mean + SD or median (Q1, Q3) and compared using mean or median differences, respectively. 95%CIs of nonparametric distribution estimated using × 1000 bootstrapping. A non-null 95% confidence interval was considered significant. Bold font are statistically significant results. Pre-COVID = patients included during March–April 2019, COVID = patients included during March–April 2020. ^a^ Observations of the 2 patients

Abbreviations: SpO_2_/ FiO_2_, oxygen saturation/fraction of inspired oxygen; CI, confidence interval; COVID, coronavirus disease

**Fig. 3:**
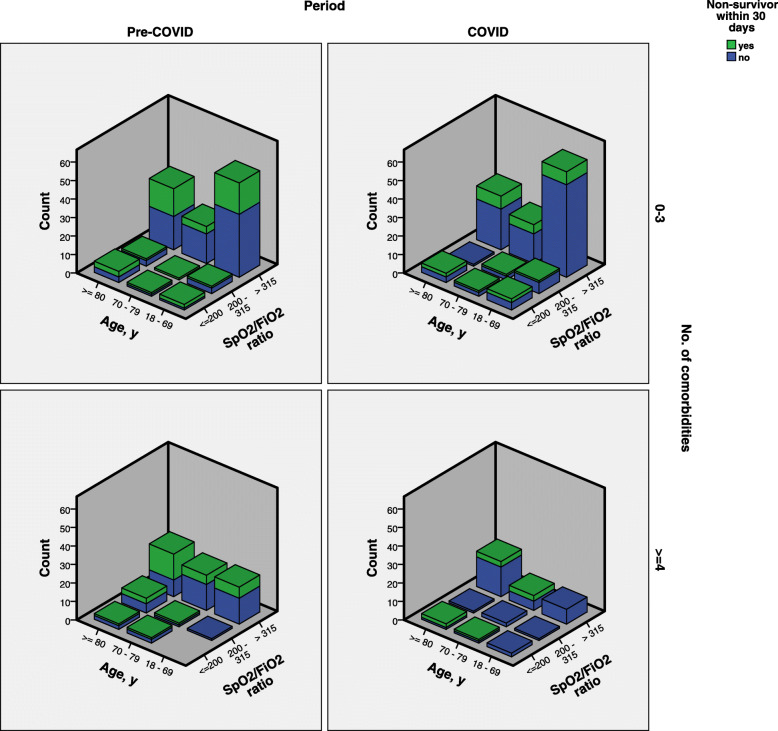
Mortality of patients with pneumonia between pre-COVID and COVID period according to age, SpO_2_/FiO_2_ strata and number of comorbidities. Abbreviations: SpO_2_/ FiO_2_, oxygen saturation/fraction of inspired oxygen

We further evaluated and compared mortality of patients with pneumonia, the majority of patients who died, based on age and SpO_2_/FiO_2_ ratio strata and number of comorbidities (Fig. 3). The distribution of age and SpO_2_/FiO_2_ ratio strata were similar among patients in the COVID and pre-COVID period, regardless of number of co-morbidities. With similar number of comorbidities, however, a higher proportion of patients in the pre-COVID died compared to the COVID period.

## Discussion

The COVID-19 outbreak has affected not only infected patients but also other non-COVID patients. There have been multiple reports of a decrease in admissions due to both critical and non-emergency conditions [13–15, 18]. Both the patients and healthcare providers have been facing unprecedented circumstances. With harder access to healthcare service, fear of being infected during hospital attendance, and possibly economic difficulty, patient visit rate has decreased dramatically during the COVID period, as also found in this study. Moreover, since they might have waited until their symptoms had progressively worsened before deciding to come to the hospital, the severity of symptoms upon presentation was significantly higher, as seen by the overall lower mean SpO_2_ and higher initial severity based on respiratory rate and SpO_2_/FiO_2_ ratio across different diagnoses in the present study. This could have been because of a generally higher severity of patients at presentation in the COVID period, despite themselves being younger with less debilitating co-morbidities.

In the present study, the rate of non-invasive airway management and nebulization, which could have generated droplets/aerosols, in non-COVID infected patients has plummeted during the COVID-19 pandemic. Furthermore, ED physicians initiated non-invasive ventilatory support when patients were more severe than usual in the elderly. This could have been because of a higher threshold to initiate these interventions due to the fear of disease transmission to healthcare providers. Our results thus confirm that the avoidance of droplet/aerosol-generating procedures also occur in non-COVID patients. Although we healthcare providers typically make our treatment decisions based on the patients’ risk and benefit, our judgement may have been altered by our own risk during an infectious disease pandemic. The situation occurred in our non-COVID ED because even though the patients had passed initial screening, ED personnel were still concerned by the uncertainty of the patients’ COVID status, as 5.7% were under-triaged and considered “at-risk” during their ED visits.

Although the failure rate of NIV and HFNC was lower in the COVID period, this could have been due to a more appropriate patient selection and clinical judgement before treatment initiation in the COVID period. Previous evidences have shown high failure rates in places where NIV was not appropriately selected and initiated [19, 20]. In addition, the lower failure rate in the COVID period could also have partly been due to the increasing trend of non-invasive airway support for palliative purposes over the past year. Recent evidence has reported that HFNC can help relieve dyspnea in DNI patients in the ED [21]; consequently, 66.7% of patients treated with HFNC in the COVID period were DNI patients as opposed to only 26.7% in the pre-COVID period. Similarly, more patients treated with NIV had DNI status in the COVID compared to the pre-COVID period (13.3 and 9.1%, respectively). In fact, a decrease in NIV and HFNC utilization could have had a direct effect on DNI patients, as they would have only been given low-flow oxygen without any ventilatory support to relieve their dyspnea.

This change of practice has affected patients across multiple diagnoses. More patients with pulmonary edema were intubated in the COVID compared to the pre-COVID period, with a trend towards a statistical significance. These results might have been partly explained by the lower rate of treatment with NIV and HFNC in the COVID period since the benefits of both non-invasive airway measures have been extensively established in this condition [5, 22]. Although the concern of viral transmission to healthcare providers has made this choice debatable in the COVID-19 era, previous evidence has demonstrated feasibility and safety of these non-invasive airway equipment [4, 8, 9, 23]. Breath dispersion distance from both NIV and HFNC was limited, given a tightly sealed interface, thereby decreasing the possibility of viral transmission [10]. Although providing and monitoring such a tight connection may not be as simple in the ED, especially for HFNC, this problem may be avoided if a mask is worn covering the HFNC. This method has shown to effectively reduce the dispersion distance during HFNC and standard nasal cannula therapy [24–26]. Nonetheless, all healthcare providers should always wear appropriate PPE with an N95 mask when providing care for a patient because there might be a risk of aerosol generation [27]. Another possible solution is to obtain a rapid test for COVID-19 in patients in whom non-invasive respiratory support is indicated. Nevertheless, in patients with obstructive lung conditions, the intubation rate in the COVID period was not higher than the pre-COVID period, despite a decrease in nebulization and NIV use. This could have also been due to a more appropriate patient selection for NIV. More interestingly, this study may provide an evidence that MDI may not be inferior to nebulization. Nonetheless, further randomized controlled trials should be conducted to confirm this hypothesis.

One clear benefit of an infectious disease pandemic found in this study was a lower rate of infectious complications, which could have led to a lower mortality rate, as seen in the present study. This lower nosocomial infection rate could have been because of the strict precaution and infectious control protocol during the pandemic. It might also have been because patients in the COVID period were younger with less severe debilitating co-morbidities, which may have also led to a lower mortality. Another possible reason for the lower mortality in the COVID period was that patients at high risk for mortality could have been treated or died at home or hospitals nearer to their residence rather than coming to a tertiary hospital in the city center during an infectious pandemic.

## Limitations

There were several limitations to the present study. Firstly, it was a single-centered study, which may have limited its generalizability to other settings. Secondly, there were no standard criteria for when to initiate each type of airway equipment, and ED attending physicians usually make their decisions, both to initiate and to escalate to a more invasive procedure, on a case-by-case basis. However, the attending physicians working on shifts during both study periods were the same group of physicians. Consequently, different airway management between the two study periods was not confounded by different physicians. Thirdly, only a single control period from the previous year was recruited in the pre-COVID-19 era, which may not be sufficient to represent the pre-COVID population. Also, the sample size of some subgroups of patients was relatively small, limiting the validity of some of the study findings. Further larger multicentered studies with predefined respiratory protocols should be conducted.

## Conclusion

In the present study, we found that the rate of non-invasive respiratory support was lower during the COVID-19 outbreak. Although not directly affecting the overall intubation rate, some groups of patients may have lost benefits from this change of practice, particularly those with pulmonary edema and DNI status, while some patients may have benefited from lower infectious complications. This study underlines another aspect of the COVID-19 pandemic, focusing on the impact that an infectious disease outbreak might have had on patients without the disease. This information can guide physicians and help plan hospital and health policy, should a similar outbreak occur in the future, as access to care, quality of care, and safety of healthcare providers are of similar importance.

## Supplementary Information

**Additional file 1 Table S1** Propensity score balance by standardized mean difference

## Data Availability

The dataset is not available but can be requested from the corresponding author.

## Abbreviations

COVID-19: coronavirus disease 2019
NIV: noninvasive ventilation
HFNC: high-flow oxygen via nasal cannula
PPE: personal protective equipment
ED: emergency department
ID: infectious disease
PUI: Patient Under Investigation
SpO2: pulse oximetry reading
COPD: chronic obstructive pulmonary disease
HAP: hospital-associated pneumonia
VAP: ventilator-associated pneumonia
CCI: Charlson comorbidity index
DNI: do-not-intubate
MDI: metered-dose inhaler
