# Supplementary material for: The COVID-19 pandemic: the effect on airway Management in non-COVID emergency patients
Source: BMC Emerg Med. 2021 Aug 28;21:97. doi: 10.1186/s12873-021-00491-7 (PMC8397873; doi:10.1186/s12873-021-00491-7)
Supplement: Supplementary file 1 — Additional file 1 Table S1 Propensity score balance by standardized mean difference [file 12873_2021_491_MOESM1_ESM.pdf]

**Table S1.** Propensity score balance by standardized mean difference

| Covariate | Before IPTW | After IPTW |
|---|---|---|
| Age | - 0.166 | 0.001 |
| Gender | - 0.052 | 0.001 |
| Charlson comorbidity index | - 0.196 | 0.001 |
| Respiratory rate | 0.052 | - 0.002 |
| Mean arterial pressure | 0.268 | 0.001 |
| $SpO_2/FiO_2$ ratio | - 0.131 | 0.001 |

Abbreviations: IPTW, inverse probability of treatment weighted; $SpO_2$/ $FiO_2$, oxygen saturation/fraction of inspired oxygen.
